# Computational Processing and Quality Control of Hi-C, Capture Hi-C and Capture-C Data

**DOI:** 10.3390/genes10070548

**Published:** 2019-07-18

**Authors:** Peter Hansen, Michael Gargano, Jochen Hecht, Jonas Ibn-Salem, Guy Karlebach, Johannes T. Roehr, Peter N. Robinson

**Affiliations:** 1 The Jackson Laboratory for Genomic Medicine, 10 Discovery Drive, Farmington, CT 06032, USA; 2 Institute of Medical Genetics and Human Genetics, Charité-Universitätsmedizin Berlin, Augustenburger Platz 1, 13353 Berlin, Germany; 3 Genomics Unit, Centre for Genomic Regulation, Carrer del Dr. Aiguader 88, 08003 Barcelona, Spain; 4 TRON—Translational Oncology at the University Medical Center of Johannes Gutenberg University, Freiligrathstrasse 12, 55131 Mainz, Germany; 5 Department of Mathematics and Computer Science, Institute of Bioinformatics, FU Berlin, 14195 Berlin, Germany; 6 Institute for Systems Genomics, University of Connecticut, Farmington, CT 06032, USA

**Keywords:** Hi-C, capture Hi-C, processing, quality control

## Abstract

Hi-C, capture Hi-C (CHC) and Capture-C have contributed greatly to our present understanding of the three-dimensional organization of genomes in the context of transcriptional regulation by characterizing the roles of topological associated domains, enhancer promoter loops and other three-dimensional genomic interactions. The analysis is based on counts of chimeric read pairs that map to interacting regions of the genome. However, the processing and quality control presents a number of unique challenges. We review here the experimental and computational foundations and explain how the characteristics of restriction digests, sonication fragments and read pairs can be exploited to distinguish technical artefacts from valid read pairs originating from true chromatin interactions.

## 1. Introduction

Three-dimensional folding of chromatin can bring functional elements such as promoters and enhancers into contact, even though they are widely separated in the linear sequence of the genome. Hi-C is a global method for interrogating chromatin interactions that combines formaldehyde-mediated cross-linking of chromatin with fragmentation, DNA ligation and high-throughput sequencing to characterize interacting loci on a genome-wide scale [1]. Although Hi-C has proved to be an extremely powerful method for investigating the large-scale architectural features of the genome such as topologically associating domains (TADs) [2], in most cases, the resolution of Hi-C libraries is not sufficient to investigate interactions between specific gene promoters and their distal regulatory elements [3].

The unique features of the chimeric read pairs, as well as the high frequency of artefactual pairs, complicate even the primary steps of the computational analysis. Here, we present a review of computational approaches to the processing and quality control of Hi-C, Capture Hi-C (CHC) and Capture-C data. In the first section of this work, we present an overview of experimental protocols with an emphasis on experimental parameters that are important for the computational analysis. Based on this, we discuss the main computational pre-processing and quality control steps that have to be performed before downstream analysis and give a brief overview of available tools and literature. Finally, we present the analysis of three representative Hi-C, CHC and Capture-C datasets, pointing out similarities and differences between the protocols.

## 2. Experimental Protocols: Hi-C, CHC and Capture-C

Hi-C combines formaldehyde-mediated cross-linking of chromatin with fragmentation, DNA ligation and paired-end short-read sequencing in order to identify pairwise contacts between genomic regions. Capture Hi-C (CHC) and Capture-C methodologies employ a hybridization technology similar to exome capture that enriches Hi-C libraries for viewpoint sequences representing loci of interest using biotinylated complementary RNA (cRNA) probes. The enrichment step adds a layer of complexity to the computational processing and quality control of CHC data. We refer to the original publications for details on the experimental protocols [3,4,5,6,7].

### 2.1. Cross-Linking and Digestion

**Figure 1:**
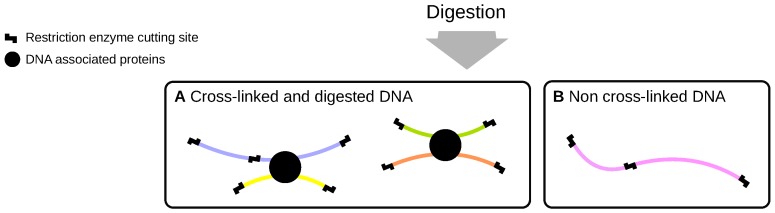
The restriction digestion of cross-linked chromatin results in fragments, also referred to as digests, whose ends correspond to restriction cutting sites of the chosen enzyme (step-like symbols). At this stage, the sample consists of a mixture of cross-linked protein-DNA complexes (**A**) and non cross-linked DNA (**B**). The digestion cannot be assumed to be complete, for instance, due to inaccessibility of DNA. Therefore, uncut restriction sites may also occur within digests.

The experimental specimens, such as cells or tissues, are first crosslinked with formaldehyde to generate covalent bonds between interacting or nearby chromatin regions. In the first step, a restriction enzyme is used in order to digest DNA that is cross-linked to the same protein or protein complex as a result of chromosomal interactions. This effectively segments the genome into a disjoint set of restriction digests defined by the enzyme (or enzyme combination). In general, it cannot be assumed that the digestion is complete and therefore digests may contain uncut restriction sites. The average size of the digests defines the lower limit of the resolution of the method (generally around 4000 bp for six-cutters such as *Hind*III and 900 bp for four-cutters such as *Dpn*II). At this stage, the sample contains a mixture of cross-linked and non cross-linked DNA digests that have sticky ends on both termini (Figure 1). For instance, the enzyme *Hind*III has the recognition sequence 5’-AAGCTT-3’ and so following restriction, the overhang at sticky ends is:

5’-A-3’

3’-TTCGA-5’

### 2.2. Ligation

For the Hi-C protocol, the sticky ends are filled in (and simultaneously labeled) with biotin-14-dATP together with unbiotinylated dCTP, dGTP and dTTP in a Klenow end-filling reaction and the resulting blunt ends are re-ligated with T4 DNA ligase. The intermediate sites that link pairs of digests are referred to as ligation junctions and the biotin labels function as baits that enable DNA fragments that arise from re-ligated digests to be enriched and un-ligated digests to be discarded. The target DNA sequence is determined by the chosen enzyme. For instance, for *Hind*III it is:

5’-AAGCTAGCTT-3’

3’-TTCGATCGAA-5’

In contrast to Hi-C, the sticky ends are not filled in and labeled with biotin for the Capture-C protocol. This results in a slightly different sequence at ligation junctions (no repetition of the overhang). More importantly, no enrichment for fragments arising from ligation can be performed.

**Figure 2:**
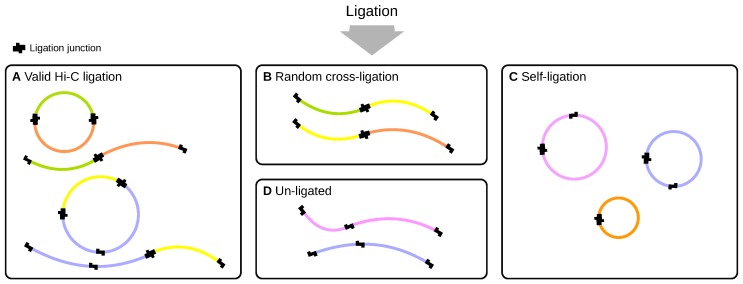
Ligation between digests within the same cross-linked protein-DNA complex results in intended chimeric Hi-C products that consist of digest pairs linked by ligation junctions. Given pairs may form circular or linear molecules (**A**). Beyond that, the ends of digests from different protein-DNA complexes may also ligate, which is referred to as random cross-ligation. Those unintentional ligations lead to false positive predicted interactions (**B**). Furthermore, the ends of individual digests may ligate, which results in circular molecules and is referred to as self-ligation (**C**). Finally, the ends of given digests may remain un-ligated (**D**).

Three types of ligation are possible. In the desired form of Hi-C ligation, interacting restriction digests attach to one another, forming either linear or circular molecules, depending on whether only one or both ends of the digests were ligated; we refer to this category as valid ligation (Figure 2A). The termini of digests from different protein-DNA complexes may also ligate, which we refer to as random cross-ligation. Those unintentional ligations can lead to false positive predicted interactions, because the random cross-ligation products cannot be distinguished from valid Hi-C products (Figure 2B). Furthermore, ligation of the two ends of individual digests may occur, which results in circular molecules and is referred to as self-ligation (Figure 2C). Finally, digests may remain un-ligated (Figure 2D). If we find a read pair that maps to two or more adjacent restriction fragments, we cannot directly observe if the read pair was the result of incomplete digestion or ligation of the adjacent restriction fragments. In either case, the resulting read pairs do not represent genuine three-dimensional interactions. We use a size threshold to classify such fragments as “un-ligated” if their length is below the threshold.

### 2.3. Shearing by Sonication

**Figure 3:**
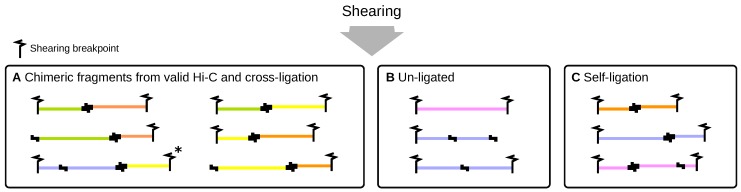
Shearing re-linearizes ring-shaped re-ligation products and introduces a new type of fragment end (denoted by flash-like symbols). At this stage, three different categories of fragments can be distinguished: chimeric fragments arising from interactions or cross-ligation (**A**) as well as fragments arising from un-ligated (**B**) and self-ligated digests (**C**). The size distribution of fragments results from digestion and shearing and can be assumed to be the same for all three categories. For chimeric fragments that contain multiple restriction sites, the size cannot be unambiguously determined (marked with an asterisk, see text below).

After the ligation step, the resulting molecules are sheared by sonication and the sonicated DNA is end-repaired. This re-linearizes the circularized ligation products. In general, the termini of sonication fragments do not coincide with restriction enzyme cutting sites. Conceptually, three different fragment categories can be distinguished at this stage (Figure 3). Chimeric fragments arising from valid ligation or random cross-ligation consist of two DNA segments from different genomic locations and are linked by a ligation junction. If both segments are located on the same chromosome, the fragments are referred to as cis and otherwise as trans. Fragments arising from un-ligated digests do not contain ligation junctions, whereas fragments arising from self-ligation do. Note that fragments without ligation junctions may also arise from digests involved in ligations because shearing of ligated digests can occasionally produce pieces of DNA without a ligation junction.

In theory, all three fragment types may contain uncut restriction sites due to incomplete digestion. If no fill in of the sticky ends was performed, ligation junctions and uncut sites have the same DNA sequence, whereas, if the fill in was performed, ligation junctions occur as two consecutive repetitions of the overhang sequence. Fragment ends that correspond to un-ligated termini of restriction digests are referred to as dangling ends. Dangling ends are most likely to occur at the ends of fragments arising from un-ligated digests, because all these digests have two un-ligated ends and sonication will inevitably produce fragments with dangling ends. Other fragment categories that may have dangling ends are chimeric fragments arising from random cross-ligation or from incomplete ligations within given DNA-protein complexes (because only one pair of ends was ligated). Finally, all ring-shaped digests are very unlikely to result in dangling end fragments unless by chance breakpoints are introduced at restriction cutting sites.

### 2.4. Sequencing and Mapping

**Figure 4:**
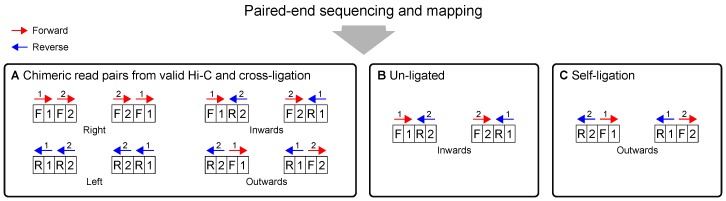
Only the two outermost ends of fragments are subjected to paired-end sequencing and mapped to the forward (red) and reverse strand (blue) of the corresponding reference genome. Read pairs arising from chimeric fragments may have all possible relative orientations (**A**). Read pairs arising from un-ligated fragments can only point inwards (**B**). Read pairs arising from self-ligation must point outwards (**C**).

For Hi-C, paired-end sequencing of the two outermost ends of fragments is performed and the reads are independently mapped (treated as single-end reads) to the corresponding reference genome on the basis of sequence identity. Since each read can map either to the positive strand (forward orientation) or to the negative strand (reverse orientation), four different relative orientations of mapped read pairs are possible (Figure 4). If both reads are mapped to the same strand, they point in the same direction, either to the left or right. If both reads are mapped to different strands, the sequential order matters and the reads point either inwards or outwards. Read pairs from chimeric fragments may have all possible orientations. In contrast, sequencing of un-ligated fragments must result in inward pointing pairs, whereas sequencing of fragments arising from self-ligation must result in outward pointing pairs.

### 2.5. Enrichment of Target Fragments

For Hi-C, an enrichment step is performed in which the biotin-marked ligation products are enriched using streptavidin Dynabeads [4]. This effectively depletes the un-ligated fragments, because all other fragment types now contain biotinylated ligation junctions. This step is not performed for Capture-C [7,8], which is why, all else equal, one has to sequence more reads from un-ligated fragments in order to obtain a comparable number of reads from chimeric fragments. Capture Hi-C and Capture-C involve an additional enrichment step using biotinylated oligonucleotides that are referred to as baits or probes and complement target regions in the genome such as promoters [6,7]. In this way, sequencing is focused on a selected set of target regions thereby reducing the sequencing depth required in order to obtain the desired coverage of the target regions. Ideally, the specific characteristics of Hi-C fragments are taken into account for bait design, which can be a challenging task for various reasons. For instance, assuming that the shearing breakpoints introduced by sonication are evenly distributed across the genome, the biotin-marked ligation junctions on chimeric fragments would accumulate around the fragment centers. In this situation, it would be sufficient (and possibly more favourable) to target only the outermost ends of digests, that is, near restriction sites. This and other challenges were addressed by GOPHER, an easy-to-use and robust desktop application for CHC probe design [9].

## 3. A Processing Pipeline for Read Pair Categorization

The processing and quality control steps can be divided into three main steps. The truncation step removes sequences from chimeric reads that would impede mapping; the alignment step maps each (potentially truncated) read separately and then rejoins the reads and determines the relative orientation of the “re-paired” reads. The pairs are then classified as artefactual or valid, and the counts of valid read pairs are determined for individual pairs of restriction digests (interactions). The resulting matrix of interactions can be used for downstream analysis.

### 3.1. Truncation of Reads

**Figure 5:**
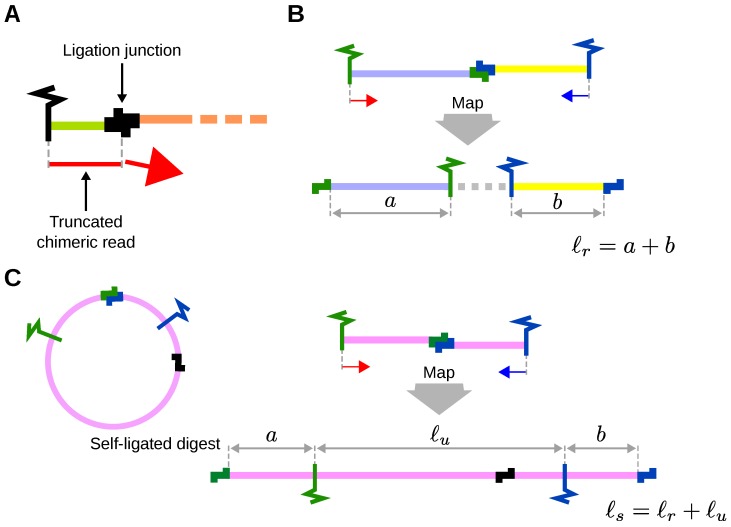
Truncation of reads and calculation of fragment and digest sizes. Ligation junctions are sought in 5’-3’ direction; reads are 3’-truncated after any idenfitifed ligation junction. (**A**). Read pairs correspond to the outermost ends of fragments. The size of ligation fragments ($l_{r}$) is calculated by summing up the sizes of the two segments that form the fragments (**B**). The size of self-ligating digests is calculated by adding the size of the un-ligated part ($l_{u}$) of the digest to the calculated fragment size (**C**).

For Hi-C, the sticky ends are filled in with biotinylated nucleotides and the resulting blunt ends are ligated. The corresponding ligation junctions can then be observed as two consecutive copies of the overhang sequence at restriction enzyme cutting sites (e.g., AAGCTAGCTT for *Hind*III; see Section 2.2). Depending on the distance of the ligation junction to the terminus of the sonication fragment, the read sequence can consist of sequences from two different digests separated by the ligation junction (Figure 5A). On average, longer read lengths and smaller size-selected fragments following sonification are more likely to produce reads that contain a ligation junction. Read mappers cannot map chimeric reads with 5’ and 3’ segments that correspond to two different genomic locations. Therefore, sequences are read in the 5’-3’ direction and chimeric reads are truncated at the location of the ligation site, thereby removing the following sequence (other strategies are also in use [10]). In contrast to Hi-C and CHC, no fill in of the overhangs is performed for Capture-C and the ligation junctions occur as plain restriction sites but the truncation step is performed in an analogous fashion.

### 3.2. Independent Mapping of Reads and Re-Pairing

The digestion and ligation steps of the Hi-C protocol require each read of a given pair to be mapped separately. A read mapper such as bowtie2 [11] can be used in single-end mode to map the truncated forward and reverse reads independently. Information about the relative order and orientation needs to be combined subsequently (“re-paired”) [12], which results in the four different read pair orientations: left, right, inwards and outwards (Figure 4). Mapped reads are stored in the SAM format [13], which allows every possible relative orientation to be represented with SAM flags.

### 3.3. Fragment and Digest Size Calculations

In order to decide whether a given read pair originates from a chimeric, un-ligated or self-ligated fragment, thresholds are applied to fragment and digest sizes. For the determination of these sizes, the special characteristics of Hi-C data must be taken into account.

The size of un-ligated fragments is only defined for inward pointing read pairs that map to the same chromosome (cis) and corresponds to the distance between the 5’ end positions of the two mapped reads, as usual. The size of fragments arising from ligation is defined for all read pairs and calculated by summing up the sizes of the two segments that form a fragment. The size of the individual segments corresponds to the distance between the 5’ end position of a mapped read and the next occurence of a restriction site in 3’ direction (Figure 5B).

Another relevant size is that of self-ligating digests which is only defined for outward pointing read pairs mapping to the same chromosome and corresponds to the genomic distance between the two restriction sites that re-ligated. This size can be calculated by adding the size of the un-ligated part of the self-ligated digest that corresponds to distance between the two 5’ end positions of the mapped reads to the calculated fragment size (Figure 5C).

We note that the size calculation procedure for fragments arising from ligation does not take into account incomplete digestion, that is, the next occurrence of a cutting site in 3’ direction does not necessarily correspond to a ligation junction (fragment marked with an asterisk in Figure 3). It is impossible to determine with certainty whether incomplete digestion or an interaction has occurred. In such cases the calculated fragment size will be shorter than the actual size.

### 3.4. Elimination of Artefactual Read Pairs

**Figure 6:**
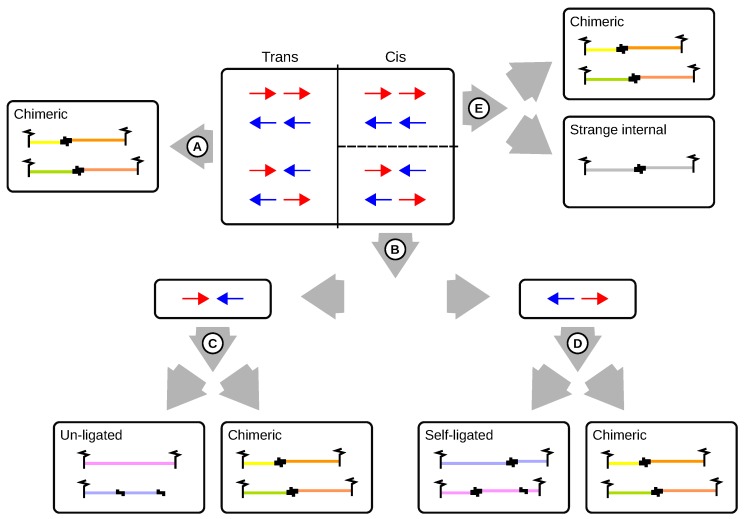
Processing logic for read pair filtering. Trans reads by definition are chimeric fragments and may represent valid biological interactions or random cross-ligation events (**A**). Pairs mapping to different strands of the same chromosome may originate from un-ligated or self-ligated digests (**B**). Inward pointing pairs that map to the same digest must have originated from un-ligated fragments. A size threshold is applied to the remaining fragments to categorize them as valid or artefactual (**C**). Outward pointing read pairs that map the same digest must have originated from self-ligated digests. A second size threshold is applied to the remaining fragments to categorize them as valid or artefactual (**D**). Read pairs mapping to the same strand can only be chimeric. However, we observe very small proportions of read pairs that are mapped to the same strand and digest. Such read pairs are classified as strange internal (**E**).

Read pairs that originate from un-ligated or self-ligated digests are not informative and need to be filtered out. The processing of Capture-C, CHC and Hi-C data is based on read pair orientation and thresholds that are applied to the sizes of fragments and self-ligated digests (Figure 6).

Read pairs that map to different chromosomes obviously originate from chimeric fragments. Furthermore, read pairs can be distinguished by means of their relative orientation. Pairs mapping to different strands of the same chromosome may be valid or originate from cross-ligated, un-ligated or self-ligated digests. Pairs where both reads map to the same restriction digest are clearly artefactual. If the read pair points inwards, then the fragment is classified as un-ligated. If the read pair points outwards, the fragment is classified as self-ligated (Figure 2 and Figure 4). If a read pair maps to two adjacent fragments, then in principle this could represent a short-range interaction or could result from incomplete digestion of an un-ligated fragment or ligation of adjacent restriction digests. It is impossible to experimentally distinguish between these possibilities. A threshold is applied to the size of un-ligated fragments ($l_{u}$; Figure 5B). If $l_{u}$ is within the expected range for fragments after shearing (not longer than a few hundred base pairs), the read pair is classified as un-ligated even if the reads are mapped to adjacent intervals that are flanked by different restriction sites. Read pairs that encompass multiple adjacent restriction fragments but whose size is below the threshold are also classified as artefacts.

A second threshold is applied that relates to the original size of self-ligating digests ($l_{s}$; Figure 5C). This size corresponds to the distance between the two restriction sites that define the self-ligating digest. If this size is within the expected range for self-ligating digests (not longer than a few thousand base pairs), a read pair is classified as self-ligated.

In contrast to un-ligated and self-ligated pairs, read pairs mapping to the same strand can only be chimeric. However, a very small proportion of read pairs (less than 0.1%) can be observed to be mapped to the same strand and to the same restriction digest. These pairs, which we refer to as strange internal because they do not correspond to any of the categories discussed above, presumably represent technical artefacts.

For the remaining chimeric read pairs, a third threshold is the ligation size ($l_{r}$). If this size is outside the expected range for fragments after shearing the corresponding read pairs are classified as too short or too long. All other chimeric read pairs are classified as valid and are suitable for downstream analysis.

We note that the chimeric read pairs with a valid size contain an unobservable but presumably large proportion of read pairs that originate from random cross-ligations. Such read pairs cannot be eliminated by the quality-control pipeline because they are indistinguishable from read pairs arising from genuine interactions.

### 3.5. Quality Metrics

It is important to understand how experimental procedures affect the values of the metrics in order to interpret them in the context of new experiments. A variety of counts and proportions can be useful in assessing the quality of an experimental dataset. Quality metrics are derived for the three major steps of the processing pipeline as well as for the overall result of the experiment (Table 1). It is not currently possible to define thresholds above or below which a dataset must be regarded as being low or high quality. Instead, we recommend that these quality metrics be compared for individual experiments to identify outliers or failed experiments that might need to be repeated or omitted from further analysis.

**Figure 7:**
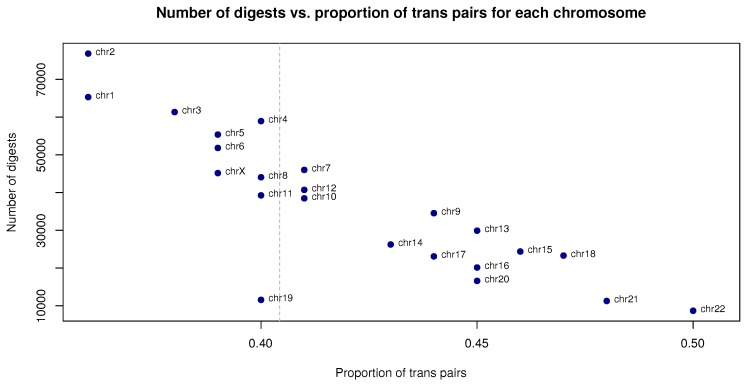
Proportion of trans read pairs per chromosome vs. total number of restriction digests per chromosome for a Capture Hi-C (CHC) experiment in human GM12878 cells (ERR436026).

Following truncation and mapping, read pairs are categorized according to fragment size and orientation and the resulting assignments of read pairs to the artefact categories or to chimeric read pairs of valid size is reported. All trans read pairs must be chimeric because they map to different chromosomes. Trans read pairs in principle may represent genuine interchromosomal interactions but trans read pairs are enriched in artefactual interactions and high trans/cis ratios may be indicative of poor library quality [12,14]. This interpretation is supported by the fact that the proportion of trans read pairs compared to all read pairs that map to a chromosome is approximately linearly related to the number of digests per chromosome (with the largest chromosomes such as chr1 and chr2 having substantially fewer trans pairs than small chromosomes such as chr21 and chr22) (Figure 7).

## 4. Computational Tools for Processing Hi-C and Capture Hi-C Data

Many authors have presented tools for processing Hi-C data that implement the strategies discussed above or variations thereof. HiC-Pro [15], Juicer [16], HiCUP [12], HiCdat [17], HOMER [18] and HiC-bench [19] are some of the best known tools.

This review is focused on pre-processing and quality control. However, we will briefly summarize typical computational analysis procedures following the pre-processing. The goal of most experiments is to determine characteristic interactions between genomic regions. The analysis can be carried out on individual restriction fragments but especially for Hi-C, interactions are often combined into genomic bins of fixed size (e.g., 5 kb, 20 kb, 40 kb, *…*, 1 Mb). The counts of chimeric loci stemming from different genomic regions reflect the strength of the genomic interactions between them. However, factors including the distance between restriction sites, the GC content of the fragments and sequence uniqueness introduce systematic biases that can affect interpretation [20] and therefore it is desirable to normalize the raw counts prior to downstream analysis [21,22]. Multiple approaches are used to normalize raw read count data, including Poisson regression [22], negative binomial regression [23], iterative correction and eigenvector decomposition [15,24,25], locally weighted linear regression of multiple datasets [21] and others.

A multitude of downstream analysis methods exist and have been reviewed elsewhere [10,26,27,28,29,30]. Prominent use cases include the detection of genomic compartments [4] or topologically associating domains (TADs) [31,32], characterizing significant chromatin interactions in Hi-C or CHC data [23,33,34], identifying copy number variations and translocations in cancer data [35] and characterizing chromatin interactions that are associated with genome-wide association study risk loci [36].

## 5. Three Exemplary Datasets

To illustrate the analysis strategy and introduce the quality metrics on real data we applied our processing pipeline and quality control analysis (Diachromatic, see Methods) to a Capture-C [8], a CHC and a Hi-C dataset [37] (See Methods for a description of the datasets). The resulting quality metrics are shown in Table 1.

If the truncation removes too much of one read for it to be reliably mapped, then the read pair is removed from further analysis. The proportion of reads removed following truncation is much higher for the Capture-C dataset than for the other two datasets (roughly 3% for Hi-C and 20% for Capture-C). Presumably, this reflects the fact that the Capture-C experiments were performed with *Dpn*II (GATC) and the other two datasets were performed with *Hind*III digestion followed by biotin fill in, which results in a much longer ligation sequence (AAGCTAGCTT) that is less likely to occur by chance. The alignment step performed by Diachromatic uses bowtie2 [11] and records the number of reads that could not be mapped or were multimapped; both categories of pairs are omitted from further analysis. Finally, successfully re-paired reads are examined for duplicates and the duplicates are removed. The target enrichment tends to reduce the complexity of the library and so the proportion of duplicates is higher for the capture Hi-C and Capture-C libraries (about 3% for Hi-C and 12–30% for CHC and Capture-C). In these datasets, roughly between 40% and 60% percent of read pairs were then available for further analysis. About 3–4% of read pairs in the experiments analyzed here contained at least one read with a dangling end. In Diachromatic, dangling read pairs are not removed because they may be the result of incomplete ligation (see Section 2.3).

The fact that there are more self-ligated read-pairs in the capture Hi-C library as compared to the Capture-C library probably reflects the fact that the biotin pull down enriches all categories of read pairs with ligation junctions (including self-ligated pairs) and tends to deplete un-ligated read pairs (which were more frequent in the Capture-C dataset). The proportion of trans pairs is the highest for Hi-C. Furthermore, the proportion of read pairs that map to non-singleton interactions is higher for Capture-C and capture Hi-C as compared to Hi-C, presumably because of the enrichment step. Global quality metrics can be used to compare related experiments. The Yield of chimeric pairs (YCP) is defined as the percentage of raw read pairs that pass all quality filters and thereby are classified as valid chimeric pairs for downstream analysis. The YCP reflects the overall efficiency of the Hi-C protocol. Valid read pairs arising from genuine chromatin-chromatin interactions between different chromosomes cannot be distinguished from those arising from cross-ligation events. However, based on the assumption that random cross-ligations between DNA fragments of different chromosomes (trans) are more likely to occur than cross-ligations between DNA fragments of the same chromosome (cis), a low ratio of the numbers of cis and trans read pairs is taken as an indicator of poor Hi-C libraries [12,14]. The fact that the cis:trans ratio is higher for the Capture-C and CHC datasets probably reflects the enrichment of targeted cis interactions (the assumption here is that true interactions are more likely to be cis than trans). The non-singleton index (NSI) is simply the percentage of non-singleton read pairs among all valid chimeric read pairs. It is higher for Capture-C and CHC than for Hi-C, presumably because the enrichment step causes reproducible interactions to be sequenced multiple times. An increased amount of random cross ligation would reduce the NSI, all else equal, because it is unlikely that the same cross-ligation event occurs more than once by chance. The target enrichment coefficient (TEC) is the proportion of read pairs for which at least one of the two reads maps to a digest that was selected for target enrichment. In the experiments analyzed here, the capture Hi-C and Capture-C methodologies yield comparable results between 22% and 30%.

## 6. Conclusions

Hi-C, CHC and Capture-C are used in a broad variety of experimental settings to characterize topological associating domains and functional interactions of promoters with distal regulatory elements such as enhancers. The results of the analysis can be used to understand the effects of single nucleotide polymorphisms (SNPs) and structural variants on gene regulation and chromosomal architecture and for the analysis of gene regulatory programs in development and disease [28]. We showed that a deep understanding of the data and potential quality issues are essential for the correct interpretation of the experimental results. With the appropriate computational analysis, noise from experimental artefacts is separated from the real signal in order to identify true interactions and reconstruct the three-dimensional folding structure of genomes.

## 7. Methods

### 7.1. Datasets

We analyzed representative Capture-C, capture Hi-C and Hi-C datasets to generate the data in Table 2. The read length was 100 bp for all the datasets analyzed here.

The Capture-C dataset [8] captured 446 limb-associated gene loci in mouse at three developmental time stages in forelimb, hindlimb and midbrain. Each experiment was performed in two biological replicates. We analyzed data for 12 (SRR3950556, SRR3950558–SRR3950568) out of 14 experiments (data for two replicate experiments in fore and hindlimb were not available at the Sequence Read Archive [38] at the time of this writing). A total number of 1,123,921,557 read pairs were extracted.

For the capture Hi-C study [37], 22,000 promoters in human CD34 and GM12878 blood cells were captured. The capture Hi-C experiments were performed in two biological replicates for CD34 and in three biological replicates for GM12878 cells. For most biological replicates there are also technical replicates. Altogether, data from 9 runs are available comprising 1,308,468,350 read pairs (ERR436025–ERR436033). We did not pool technical replicates but analyzed them separately. In addition, Hi-C experiments from the same experiment [37] were analyzed. One replicate each was performed for CD34 and GM12878. The two datasets comprise 351,972,837 read pairs (ERR436023, ERR436024).

### 7.2. Diachromatic

Diachromatic is a Java application that implements the above processing and quality control pipelines. As input, Diachromatic expects paired FASTQ files from a Hi-C, CHC, or Capture-C experiment. Furthermore, Diachromatic requires a digest file as input that contains the coordinates of all digests that result from complete digest of the entire genome using a given restriction enzyme or set of enzymes. Beyond that, the digest file contains information about each digest such as length and GC content. The digest map can be generated using GOPHER [9]. Diachromatic produces a BAM file containing valid chimeric read pairs designed for downstream analysis. Diachromatic source code and complete documentation are available at the Diachromatic GitHub page (https://github.com/TheJacksonLaboratory/diachromatic).

### 7.3. Digest Map for Andrey et al. 2016

In order to create the digests map that is required as input for Diachromatic we used GOPHER (v0.5.9). The 446 gene symbols of the target genes were extracted from Table S1 of the original publication [8]. After manual revision of gene symbols that could not be found in RefSeq annotation, 433 gene symbols were imported into GOPHER. For these gene symbols we derived viewpoints for mm10 using GOPHER’s extended approach with 5000 bp in up- and 2000 bp in downstream direction. The restriction enzyme was set to *Dpn*II. This approach is similar to that taken by Andrey et al. Thresholds for GC and repeat content as well as balanced margins were overridden. Altogether, the design consists of 433 genes, 577 viewpoints, 6402 unique digests and 12,804 probes.

### 7.4. Digest Map for Mifsud et al. 2015

To create the digest map for the analysis of the data of Mifsud et al., we used GOPHER’s preset option for all protein coding genes and the simple approach with *Hind*III in order to create viewpoints for hg38. Thresholds for GC and repeat content as well as balanced margins were overridden. This results in a design with 18,957 genes, 31,832 viewpoints, 19,873 unique digests and 39,746 probes. 

## Figures and Tables

**Table 1:** Quality metrics that can be used to assess the quality of an experimental dataset. It is not possible to provide precise cutoffs for the quality metrics but instead we recommend that researchers use the metrics to compare experiments in a given study to identify potential outliers that may require attention.

Truncation, Mapping and Deduplication	
Total raw read pairs	Input read pairs in the raw FASTQ (usually $∼10^{8}-10^{9}$).
Removed by truncation	Read pairs removed because at least one of the reads was too short to map following truncation at a ligation sequence. Depends on the specificity of the read sequence at ligation junctions, which is typically higher with longer restriction enzyme recognition sequences and if sticky ends are filled in.
Unmapped/multimapped	Read pairs removed because at least one of the reads could not be mapped (or could not be mapped uniquely).
Duplicated	Removed duplicated read pairs (one pair is retained for downstream analysis). High duplication rates indicate low library complexity that may be due to low amounts of DNA used for library preparation.
Dangling ends	Read pairs at least one of whose 5’ ends coincides with a restriction enzyme cutting site. Dangling ends may correspond to un-ligated digest ends.
Remaining pairs	Total read pairs that were not removed in the course of truncation, mapping and deduplication (usually on the order of $10^{8}$).
**Re-paired read pairs**	
Un-ligated	Large proportions of un-ligated read pairs indicate inefficient biotin pull down of fragments with ligation junction. For Capture-C, the proportion of un-ligated pairs is much higher because no pull down is performed.
Self-ligated	Self-ligation seems to be a rare event. Because fragments arising from self-ligation contain ligation junctions the proportions may be higher for capture Hi-C (CHC) as compared to Capture-C.
Strange internal	Number of read pairs for which both reads map to the same strand and restriction digest. Cannot be explained by un-ligated or self-ligated digests. Typically, this category make up only very small proportions (less than 0.1% of re-paired pairs).
Chimeric	Read pairs that arise from interactions or random cross-ligations (on the order of $10^{7}$).
**Chimeric read pairs**	
Trans	Chimeric read pairs whose reads map to different chromosomes. Large proportions indicate a high degree of random cross-ligation.
Cis	Chimeric read pairs whose reads map to the same chromosome.
Non-singleton index (NSI)	Number of interactions that consist of more than one read pair. A high proportion of singleton interactions may indicate a high degree of random cross-ligation because random cross-ligations for a given digest pair are unlikely to occur more than once.
**Global quality metrics**	
Yield of chimeric pairs (YCP)	Percentage among input read pairs that are classified as chimeric and used for downstream analysis. Low percentages may indicate low overall performance of the protocol.
cis:trans ratio	Low percentages of cis read pairs indicate a high degree of random cross-ligation.
Yield of non-singleton pairs	Percentage among input read pairs that belong to interactions with more than one read pair.
Target enrichment	Percentage of chimeric read pairs for which at least on read is mapped to a target region. Low percentages indicate poor performance of target enrichment.

**Table 2:** Average read pair counts and quality metrics for Capture-C, CHC and Hi-C datasets. The percentages for truncation, mapping and deduplication were calculated with respect to the total number of read pairs. The percentages for read pair categorization were calculated with respect to the number of reads that could be re-paired (remaining reads from the first processing steps). Percentages of cis and trans read pairs as well as read pairs in non-singleton interactions were calculated with respect to the total number of pairs that were categorized as chimeric. See Table 1 for explanation of the quality metrics.

Item	Capture-C	Capture Hi-C	Hi-C
Dataset/N samples	Andrey/12	Mifsud/9	Mifsud/2
**Truncation, Mapping and Deduplication**			
Total raw read pairs	93,660,130	145,385,372	175,986,419
Percentage	100%	100%	100%
Removed by truncation	18,260,618	3,619,477	6,789,444
Percentage	19.50%	2.49%	3.86%
Unmapped/multimapped	10,265,988	41,969,433	58,468,465
Percentage	10.96%	28.87%	33.22%
Duplicated	28,963,777	17,906,644	3,467,125
Percentage	30.92%	12.32%	1.97%
**Remaining pairs**	**36,169,746**	**81,889,818**	**107,261,386**
**Percentage**	**38.62%**	**56.33%**	**60.95%**
Dangling ends	1,532,317	3,479,723	3,248,438
Percentage	4.24%	4.25%	3.03%
**Categorization of re-paired read pairs**			
Un-ligated	30,847,302	9,610,217	15,045,042
Percentage	85.28%	11.74%	14.03%
Self-ligated	108,714	2,096,818	5,290,287
Percentage	0.30%	2.26%	4.93%
Strange internal	1039	71,356	73,257
Percentage	0.00%	0.09%	0.07%
**Chimeric**	**5,212,691**	**70,111,427**	**86,852,801**
**Percentage**	**14.41%**	**85.62%**	**80.97%**
**Analysis of chimeric read pairs**			
Trans	2,359,391	34,795,199	55,458,895
Percentage	45.26%	49.63%	63.85%
Cis	2,853,300	35,316,228	31,393,906
Percentage	54.74%	50.37%	36.15%
Non-singleton index (NSI)	607,086	11,211,773	8,413,573
Percentage	11.65%	15.99%	9.69%
**Global quality metrics**			
Yield of chimeric pairs (YCP)	5.57%	48.22%	49.35%
cis:trans ratio	1.21	1.01	0.57
Yield of non-singleton pairs	0.65%	7.71%	4.78%
Target enrichment	29.91%	22.44%	n/a
